# Validation of automatic measurement of QT interval variability

**DOI:** 10.1371/journal.pone.0175087

**Published:** 2017-04-12

**Authors:** Peter R. Rijnbeek, Marten E. van den Berg, Gerard van Herpen, Henk J. Ritsema van Eck, Jan A. Kors

**Affiliations:** Department of Medical Informatics, Erasmus University Medical Center, Rotterdam, The Netherlands; University of Minnesota, UNITED STATES

## Abstract

**Background:**

Increased variability of beat-to-beat QT-interval durations on the electrocardiogram (ECG) has been associated with increased risk for fatal and non-fatal cardiac events. However, techniques for the measurement of QT variability (QTV) have not been validated since a gold standard is not available. In this study, we propose a validation method and illustrate its use for the validation of two automatic QTV measurement techniques.

**Methods:**

Our method generates artificial standard 12-lead ECGs based on the averaged P-QRS-T complexes from a variety of existing ECG signals, with simulated intrinsic (QT interval) and extrinsic (noise, baseline wander, signal length) variations. We quantified QTV by a commonly used measure, short-term QT variability (STV). Using 28,800 simulated ECGs, we assessed the performance of a conventional QTV measurement algorithm, resembling a manual QTV measurement approach, and a more advanced algorithm based on fiducial segment averaging (FSA).

**Results:**

The results for the conventional algorithm show considerable median absolute differences between the simulated and estimated STV. For the highest noise level, median differences were 4–6 ms in the absence of QTV. Increasing signal length generally yields more accurate STV estimates, but the difference in performance between 30 or 60 beats is small. The FSA algorithm proved to be very accurate, with most median absolute differences less than 0.5 ms, even for the highest levels of disturbance.

**Conclusions:**

Artificially constructed ECGs with a variety of disturbances allow validation of QTV measurement procedures. The FSA algorithm provides highly accurate STV estimates under varying signal conditions, and performs much better than traditional beat-by-beat analysis. The fully automatic operation of the FSA algorithm enables STV measurement in large sets of ECGs.

## Introduction

The duration of the QT interval in the electrocardiogram (ECG) may vary between individual beats, reflecting beat-to-beat changes in ventricular depolarization and repolarization [1]. A recent position paper about QT-interval variability (QTV) extensively reviewed the measurement, physiological basis, and clinical value of QTV [2]. Increased QT-interval variability (QTV) has been associated with increased risk for arrhythmias and cardiovascular events in general [2, 3].

The measurement of QTV is a challenging task because the QT-interval variations are usually subtle, in the order of milliseconds, and noise or baseline wander may further complicate the determination of the end of the T wave, which in itself is ill-defined. QT intervals have been measured manually, which is time-consuming and cumbersome. Alternatively, several (semi-)automatic techniques have been proposed [2], but little is known about their measurement accuracy. Validation of manual or automatic measurement techniques, preferably under different operating conditions, is needed. However, validation is equivocal because no reference standard is available.

This issue was in part addressed by Baumert et al. [4], who constructed artificial ECGs by concatenating a single, noise-free ECG beat, and then added various forms of simulated disturbances (noise, baseline wander, amplitude modulation). The simulated ECGs were then used for testing the performance of three QTV measurement algorithms. These authors did not simulate beat-to-beat QT-interval variations, and thus could only validate the performance of the algorithms in the absence of QTV. Moreover, all simulated ECGs were based on just one ECG beat from a single lead.

Here we present a validation method that generates artificial standard 12-lead ECGs based on the averaged P-QRS-T complexes from a variety of existing ECG signals, with simulated intrinsic (QT interval) and extrinsic (noise, baseline wander, signal length) variations. Using the simulated ECGs, we assessed the performance of two fully-automatic QTV measurement algorithms, viz. a conventional QTV measurement algorithm, resembling a manual QTV measurement approach, and a more advanced algorithm based on the fiducial segment averaging technique [5].

## Methods

Our validation approach consists of the following steps. First, low-noise artificial ECGs of different durations are constructed from a collection of 12-lead ECGs, and initial QT intervals of the individual beats in each artificial ECG are set. Various amounts of intrinsic variability (QTV) and extrinsic variations (noise and baseline wander) are simulated and added to the artificial ECGs. Second, the artificial ECGs are processed by a QTV measurement program and the computed QTV is compared with the simulated QTV to assess program performance. These steps are discussed in more detail below.

### Construction of artificial ECGs

For a given standard 12-lead ECG, we constructed an artificial ECG by computing an averaged P-QRS-T complex for each lead and concatenating this single complex at the same heart rate as in the original ECG. Since the complexes of the artificial ECG are per lead exactly identical, there is no QTV.

To determine the averaged complex, we had recourse to the Modular ECG Analysis System (MEANS). This program for automatic ECG measurement and diagnosis has been evaluated extensively, both by its developers and by others [6–8]. For each lead, MEANS performs baseline correction, removes mains interference, and determines an averaged complex from the dominant beats after having excluded ectopic beats. This results in a low-noise representative complex without baseline wander. MEANS determines global fiducial points in the averaged beats of all 12 leads, resulting in a common P onset, P end, QRS onset, QRS end, and T end over all leads. The fiducial points determined by MEANS are transferred to each beat in the artificial ECG, and serve as the reference points for subsequent evaluation of the QTV measurement algorithms.

### Simulation of intrinsic and extrinsic variations

**Fig 1:**
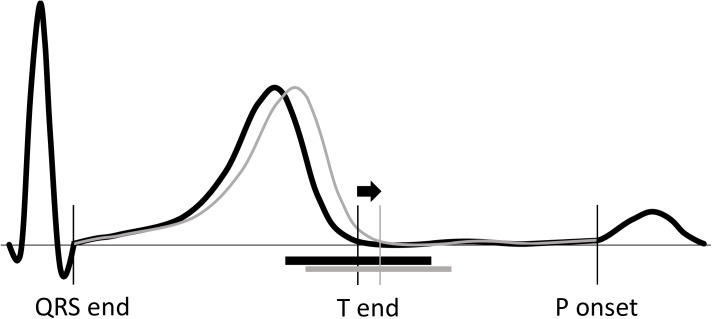
Example of a simulated QT-interval change. The black line indicates the original ECG signal with the vertical line denoting the end of the T wave as determined by the MEANS program. The grey line indicates the signal with a shifted end of the T wave. The horizontal bars below the signals mark symmetric windows of 180 ms around the end of the T wave in which the signal is not deformed. The signal segment from QRS end till the start of the window is extended, whereas the signal segment from the end of the window till the onset of the next P wave is compressed.

Assuming that QTV is mainly determined by ventricular repolarization, we simulated QT interval changes by stretching or compressing the ST-T wave of complexes, effectively shifting the end of the T wave. We did not change the onset of the QRS complex. The end of the T wave as determined by MEANS was taken as starting point. Simulated changes in the end of the T wave always consisted of an integer number of sample points (sampling interval 2 ms). A symmetric window of 90 sample points around T end was shifted in time foreward or backward without deformation, bringing about a compression or extension of the signal segments before and after the window (see Fig 1). The samples in the T wave before this window were shifted proportionally in time, interpolated, and resampled at the original sampling frequency (500 Hz). Similarly, the samples after the window till the start of the next P wave were shifted, interpolated, and resampled. For a given complex, the shift in T end was the same across all leads.

We quantified QTV by a commonly used measure, short-term QT variability (STV), which is defined as the mean absolute difference between successive QT intervals [9]:

$$STV=\sum_{i=1}^{N}\frac{\left|{QT}_{i+1}-{QT}_{i}\right|}{N\sqrt{2}}$$

To simulate a particular STV value for a signal consisting of N+1 beats, we generated a sequence of N absolute QT-interval differences (i.e., |*QT*_*i*+1_ − *QT*_*i*_|) by drawing from a uniform distribution centered around the required STV value, with a minimum of 0 and a maximum of twice the required STV. If the absolute difference between the STV of the sequence and the required value was greater than 0.1 ms, the sequence was rejected and a new sequence was generated. This was repeated until the difference was ≤ 0.1 ms. The QT durations of the individual beats were then derived from the generated QT differences, taking for the first beat the original QT interval as determined by MEANS. To avoid an ever-increasing QT interval, each (absolute) difference was added to or subtracted from the preceding QT interval so that the cumulative sum of the (signed) differences was minimized.

**Fig 2:**
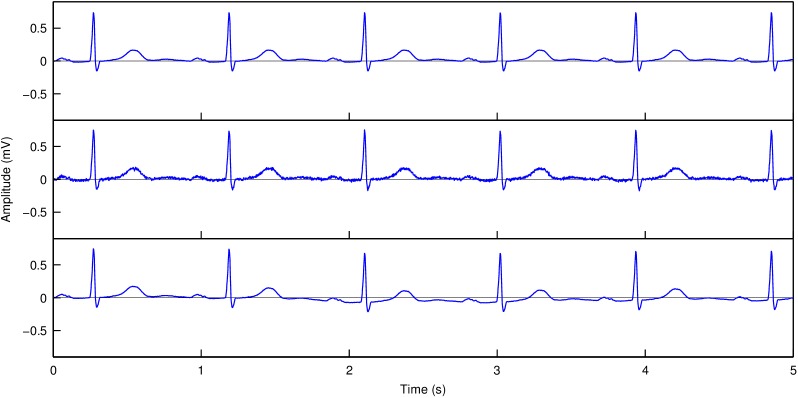
Example of simulated extrinsic disturbances. Top panel: artificial ECG signal constructed by concatenating the averaged P-QRS-T complex of the original ECG. Middle panel: artificial signal with added noise (SNR 20). Bottom panel: artificial signal with added residual baseline wander (standard deviation of slope 30 μV/s).

Two types of extrinsic variation were simulated, muscle noise and baseline wander (see Fig 2). To simulate muscle noise, we generated white noise. For each lead, this noise was added after scaling of the noise amplitude to a prespecified signal-to-noise ratio (SNR). Baseline wander was simulated by piecewise linear baseline shifts, where each piece started at the onset of a QRS complex and ended at the onset of the next QRS complex. The slope of each piece of baseline shift was randomly selected from a normal distribution with a prespecified standard deviation and zero mean. Since the simulated baseline wander might easily be removed by an automatic correction method, we chose to simulate small baseline shifts that were considered to constitute the residual baseline wander that remained after a (hypothetical) baseline correction algorithm was applied. Since small simulated pieces of baseline wander may add up to a large baseline shift if successive pieces have slopes with the same sign, we applied the following rule: if the simulated baseline amplitude at the end of a particular complex was positive, the slope of the next piece of baseline was taken negative, and vice versa, if the baseline amplitude was negative, the slope of the next piece was taken positive.

### QT variability measurement

We assessed the performance of two fully automatic QTV measurement algorithms: a conventional method based on the processing and measurement of individual ECG beats, and fiducial segment averaging, which exploits the correlation between signal segments across beats.

#### Conventional computerized measurement

The MEANS program described above also has the option to measure each individual beat in a recording separately. We used this option to determine beat-to-beat QT interval estimates for the artificial ECGs. The baseline correction of MEANS was turned off to assess the effect of residual baseline wander on QTV measurement. The processing of individual beats by MEANS reflects a manual measurement process in which QT intervals are also measured separately.

#### Fiducial segment averaging

**Fig 3:**
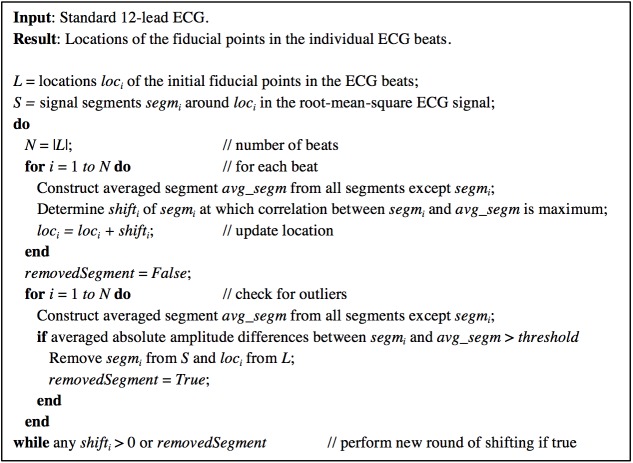
Pseudocode of the FSA algorithm.

Fiducial segment averaging (FSA) uses beat-to-beat coherence of relatively small segments within the P-QRS-T complex to improve the accuracy of fiducial point estimates. A semi-automatic version of the measurement process using FSA was first described by Ritsema van Eck [5]. In this study, we have implemented a fully automatic version (Fig 3).

First, MEANS determines the initial fiducial points (onset of QRS complex and end of T wave) and constructs a detection function consisting of the root-mean-square ECG signal [10]. Second, the fiducial point in each individual beat is shifted until maximum correlation is achieved between a 120-ms signal segment of the detection function around this fiducial point and the average of the segments around the fiducial points of all complexes. The amount of shifting is retained and constitutes the individual beat variation in the fiducial point estimate. Based on the new fiducial point estimates another round of shifting is carried out. This process is repeated until the correlations cannot be further improved. Finally, the QT interval for each beat is calculated taking into account the final shifts.

To safeguard against signal segments with excessive noise or baseline wander, the FSA algorithm applies an additional test after each round of shifting. If the averaged absolute amplitudes of the difference between the ST-T wave of an individual beat and the averaged ST-T wave of the remaining beats is larger than a preset value, the beat is discarded and the iteration process is repeated for the remaining beats. It should be noted that a rejected beat may reduce the number of QT-interval differences in the STV computation by more than one because only differences between QT intervals of consecutive beats are taken into account. Since we did not intend to simulate excessive noise or baseline wander, the number of rejected beats was expected to be negligible.

### Validation experiments

To validate the two measurement algorithms, we used the first 200 ECGs from the Common Standards for Electrocardiography (CSE) diagnostic ECG library [8]. The CSE library consists of 1,220 fully anonymized ECGs that have previously been used in various studies to assess and compare the performance of computerized ECG programs. The leads of these ECGs were recorded simultaneously at a sampling rate of 500 Hz during 10 seconds. The diagnostic classification of individual ECGs has not been released, but the database is known to contain 382 normal ECGs while the rest have various abnormalities [8].

Each of the 200 ECGs was processed by MEANS to construct averaged beats, which were used to generate artificial noise-free ECGs without QTV consisting of 10, 30, and 60 beats, as described above. For each of these ECGs, new ECGs with simulated STV values of 2, 4, 6, 8, and 10 ms were generated. For each of the resulting ECGs, further ECGs were generated by adding different amounts of noise (SNR 40, 30, or 20), residual baseline wander (standard deviation of the distribution of slopes 10, 20, or 30 μV/s), or a combination (SNR 30 and 20 μV/s baseline wander), for a total of 28,800 ECGs.

## Results

### Conventional computerized measurement

Table 1 shows the median and 95th percentile (p95) of the absolute differences between the simulated STV and the STV estimated by the conventional, beat-by-beat measurement of MEANS. For disturbance-free ECGs, the median absolute differences are in the order of 15% of the simulated STV, while p95 values are about twice as high. For low and medium noise levels (SNR 40 or 30), similar results are observed for simulated STV values of 4 ms or larger. Interestingly, the median and p95 values of the absolute differences in the absence of STV are higher than those for a simulated STV of 2 ms. This may be explained by the fact that if the simulated STV is 0, any QT-interval mismeasurement will yield an estimated STV > 0, whereas if the simulated STV is larger than 0 and QT mismeasurements are made, the estimated STV can be lower or higher, or even the same, as the simulated STV. For the highest noise level (SNR 20), performance deteriorates greatly, with median differences of 4–6 ms in the absence of STV and p95 values varying between 10 and 20 ms.

**Table 1:** Median (95th percentile) of the absolute differences between simulated STV and STV as measured by the MEANS algorithm for different signal-to-noise ratios (SNR), residual baseline wander, and number of beats.

		Simulated STV (ms)					
Artifact	No. of beats	0	2	4	6	8	10
None	10	0.00 (0.00)	0.31 (0.79)	0.63 (1.34)	1.06 (2.12)	1.26 (2.99)	1.65 (3.50)
	30	0.00 (0.00)	0.27 (0.49)	0.59 (1.02)	0.83 (1.43)	1.17 (2.23)	1.46 (2.57)
	60	0.00 (0.00)	0.26 (0.40)	0.55 (0.84)	0.83 (1.34)	1.09 (1.78)	1.40 (2.30)
SNR 40	10	1.02 (2.59)	0.31 (1.34)	0.67 (1.65)	1.02 (2.44)	1.34 (2.79)	1.69 (4.32)
	30	1.15 (2.44)	0.22 (1.05)	0.44 (1.24)	0.80 (1.77)	1.18 (2.46)	1.39 (2.97)
	60	1.20 (2.18)	0.18 (1.14)	0.42 (0.91)	0.71 (1.58)	1.05 (1.73)	1.31 (2.40)
SNR 30	10	1.49 (3.22)	0.47 (2.12)	0.79 (2.44)	1.02 (3.02)	1.22 (3.42)	1.53 (3.77)
	30	1.63 (3.91)	0.37 (2.45)	0.46 (1.27)	0.68 (1.91)	1.12 (2.35)	1.46 (3.16)
	60	1.64 (4.25)	0.43 (2.25)	0.32 (1.73)	0.66 (1.47)	0.95 (2.04)	1.27 (2.56)
SNR 20	10	4.48 (19.05)	2.36 (16.42)	1.81 (14.89)	1.57 (18.38)	1.89 (10.21)	2.12 (10.69)
	30	5.73 (18.08)	3.71 (18.28)	2.07 (14.24)	1.54 (11.29)	1.22 (14.78)	1.45 (12.19)
	60	5.82 (17.49)	3.74 (17.31)	2.40 (14.81)	1.53 (15.02)	1.10 (13.96)	1.13 (11.69)
Baseline 10 μV/s	10	0.94 (2.63)	0.39 (1.26)	0.71 (1.49)	1.02 (2.24)	1.41 (2.99)	1.65 (3.77)
	30	1.05 (2.24)	0.22 (1.13)	0.46 (1.21)	0.78 (1.71)	1.09 (2.01)	1.46 (3.06)
	60	1.03 (2.08)	0.20 (0.89)	0.47 (1.03)	0.77 (1.53)	1.06 (2.04)	1.40 (2.40)
Baseline 20 μV/s	10	1.02 (2.71)	0.39 (1.41)	0.79 (1.73)	1.02 (2.47)	1.37 (3.22)	1.57 (3.89)
	30	1.02 (2.24)	0.24 (1.27)	0.44 (1.15)	0.84 (1.55)	1.11 (2.21)	1.51 (2.91)
	60	1.07 (2.26)	0.22 (1.10)	0.46 (1.05)	0.74 (1.53)	1.08 (2.10)	1.32 (2.62)
Baseline 30 μV/s	10	1.02 (2.44)	0.39 (1.61)	0.79 (1.96)	1.10 (2.40)	1.41 (3.10)	1.73 (4.01)
	30	1.07 (2.46)	0.24 (1.68)	0.51 (1.29)	0.72 (1.84)	1.12 (2.45)	1.39 (2.84)
	60	1.10 (2.54)	0.19 (1.14)	0.41 (0.96)	0.77 (1.50)	1.04 (2.16)	1.35 (2.84)
SNR 30 +	10	1.49 (3.18)	0.47 (2.20)	0.71 (2.12)	1.02 (2.87)	1.57 (3.50)	1.73 (3.89)
baseline 20 μV/s	30	1.65 (4.17)	0.43 (2.12)	0.45 (1.66)	0.73 (1.99)	1.04 (2.33)	1.38 (2.96)
	60	1.75 (4.45)	0.36 (2.97)	0.32 (1.97)	0.71 (1.38)	0.97 (1.89)	1.33 (2.56)

Measurements are much more robust for ECGs with residual baseline wander. The absolute differences are comparable to those of slightly noisy ECGs (SNR 40). The amount of residual baseline wander hardly affects the estimates. The combination of medium noise and residual baseline (SNR 30 + slope 20 μV/s) shows similar performance as medium noise alone.

An increase in number of beats generally results in more accurate STV estimates, but the difference in performance between 30 or 60 beats is small in most cases.

### FSA measurement

Table 2 shows the median and p95 of the absolute differences between simulated and estimated STV for the FSA algorithm. For ECGs without artifacts, FSA perfectly estimates the different simulated STV values, i.e., all differences between simulated and estimated STV are zero. For ECGs with low or medium noise, most of the differences are very small (p95 well below 1 ms). For higher noise levels (SNR 20), the median absolute differences are still very small (about 1 ms for STV = 0 and less than 0.5 ms for STV > 0), while p95 values are in the range of 1–2 ms. A similar pattern with very low differences is observed for various amounts of residual baseline wander. The combination of medium noise and baseline residual gives slightly worse results than those of either artifact separately, but almost all median values remain below 0.5 ms, and most p95 values below 1 ms.

**Table 2:** Median (95th percentile) of the absolute differences between the simulated STV and STV as measured by the FSA algorithm for different signal-to-noise ratios (SNR), residual baseline wander, and number of beats.

		Simulated STV (ms)					
Artifact	No. of beats	0	2	4	6	8	10
None	10	0.00 (0.00)	0.00 (0.00)	0.00 (0.00)	0.00 (0.00)	0.00 (0.00)	0.00 (0.00)
	30	0.00 (0.00)	0.00 (0.00)	0.00 (0.00)	0.00 (0.00)	0.00 (0.00)	0.00 (0.00)
	60	0.00 (0.00)	0.00 (0.00)	0.00 (0.00)	0.00 (0.00)	0.00 (0.00)	0.00 (0.00)
SNR 40	10	0.00 (0.31)	0.00 (0.24)	0.00 (0.16)	0.00 (0.31)	0.00 (0.31)	0.00 (0.31)
	30	0.00 (0.24)	0.00 (0.10)	0.00 (0.10)	0.00 (0.10)	0.00 (0.10)	0.00 (0.15)
	60	0.00 (0.18)	0.00 (0.05)	0.00 (0.05)	0.00 (0.05)	0.00 (0.05)	0.00 (0.05)
SNR 30	10	0.47 (1.34)	0.16 (0.79)	0.31 (0.71)	0.16 (0.63)	0.16 (0.94)	0.16 (0.79)
	30	0.20 (0.93)	0.10 (0.39)	0.10 (0.39)	0.10 (0.39)	0.10 (0.39)	0.10 (0.29)
	60	0.22 (0.79)	0.05 (0.31)	0.05 (0.24)	0.05 (0.24)	0.05 (0.22)	0.05 (0.29)
SNR 20	10	1.26 (2.75)	0.31 (1.81)	0.47 (1.57)	0.47 (1.41)	0.47 (1.96)	0.47 (1.57)
	30	0.88 (2.32)	0.24 (1.02)	0.20 (1.02)	0.20 (0.95)	0.20 (1.07)	0.22 (1.19)
	60	0.77 (2.21)	0.19 (0.95)	0.14 (0.90)	0.14 (0.77)	0.17 (1.21)	0.16 (1.19)
Baseline 10 μV/s	10	0.16 (0.94)	0.00 (0.47)	0.00 (0.63)	0.00 (0.63)	0.00 (0.63)	0.00 (0.47)
	30	0.20 (1.02)	0.05 (0.34)	0.05 (0.29)	0.05 (0.29)	0.05 (0.34)	0.05 (0.29)
	60	0.19 (0.90)	0.05 (0.34)	0.05 (0.22)	0.02 (0.24)	0.05 (0.26)	0.05 (0.24)
Baseline 20 μV/s	10	0.63 (2.12)	0.16 (1.02)	0.16 (0.94)	0.16 (0.94)	0.16 (0.79)	0.16 (0.94)
	30	0.68 (1.80)	0.15 (0.76)	0.10 (0.44)	0.10 (0.49)	0.10 (0.51)	0.10 (0.59)
	60	0.67 (1.95)	0.12 (0.68)	0.10 (0.44)	0.10 (0.46)	0.10 (0.38)	0.07 (0.40)
Baseline 30 μV/s	10	0.94 (2.91)	0.31 (1.49)	0.31 (1.49)	0.31 (1.41)	0.31 (1.26)	0.31 (1.41)
	30	0.98 (2.37)	0.24 (1.24)	0.19 (0.95)	0.15 (0.88)	0.19 (0.68)	0.15 (0.78)
	60	0.96 (2.34)	0.26 (1.09)	0.13 (0.85)	0.11 (0.79)	0.10 (0.54)	0.12 (0.50)
SNR 30 +	10	0.79 (1.73)	0.31 (1.10)	0.31 (0.94)	0.31 (0.94)	0.31 (1.10)	0.31 (1.10)
baseline 20 μV/s	30	0.59 (1.41)	0.19 (0.59)	0.15 (0.49)	0.10 (0.44)	0.15 (0.49)	0.15 (0.59)
	60	0.50 (1.41)	0.10 (0.52)	0.10 (0.34)	0.07 (0.35)	0.10 (0.37)	0.10 (0.38)

The number of ECGs in which the FSA algorithm rejected beats for further analysis was very low: one ECG for the highest level of simulated noise (SNR 20), and two ECGs for the largest slope of residual baseline wander (30 μV/s).

## Discussion

We have validated the performance of two QTV measurement tools under different operating conditions by constructing artificial ECGs with different amounts of simulated STV and disturbances. Our results indicate that the FSA algorithm produces highly accurate STV estimates. A traditional beat-by-beat measurement algorithm performed less well, especially for higher levels of noise or residual baseline wander.

We are not the first to use simulated data as a means to validate the performance of QTV measurement algorithms [4, 11]. Baumert et al. [4] concatenated a noise-free beat of one ECG lead and added different forms of artifacts to validate several (semi-)automatic measurement techniques. The same data were also used in a later study, in which the authors evaluated an alternative measurement approach [11]. Beat-to-beat QT-interval variations were not simulated, and thus the performance of the algorithms was only validated in the absence of QTV. Moreover, all simulated ECGs were constructed from just one ECG beat from a single lead. We used a set of 200 different artificial 12-lead ECGs, and also simulated different amounts of STV. Contrary to the previous studies, this allowed us to validate the performance of measurement algorithms for non-zero STV values, in a morphologically diverse set of ECGs.

The same approach that we applied to validate automatic algorithms, could, in principle, also be used to validate a manual measurement procedure. We did not attempt to do this since the effort of measuring individual QT intervals in thousands of ECGs was considered prohibitive. However, the MEANS algorithm, like the manual method, also measures on a beat-by-beat basis. Our results clearly indicate that this beat-by-beat measurement is inferior to an approach that exploits the correlation between individual beats, as is done in FSA. In particular for larger noise levels, the errors in the MEANS estimates become unacceptably large. This suggests that STV estimates obtained with a beat-by-beat measurement procedure, automatic or manual, must be interpreted cautiously.

Previous studies that used STV have measured QT intervals in 30 or 60 consecutive beats [9, 12], but the effect of varying recording durations on the accuracy of STV estimates has not been investigated. Our results indicate that accuracy generally improves with increasing signal length. This effect is more pronounced for FSA than for MEANS, likely because FSA employs an averaged signal segment that will become less noisy with increasing signal length, whereas MEANS does not use averaging when measuring individual beats. We also found that FSA already performs very well for signal durations of 10 s. This finding increases the practical utility of STV as the far majority of ECGs that are recorded in clinical practice or epidemiological studies are standard 10-s ECGs. The ability to process large sets of ECGs also allows to quantify circadian effects and establish normal values of QTV, as recommended in a recent QTV position paper [2].

In this study we have focused on the validation of STV measurement. The same approach can be used to validate the measurement of other QTV parameters, such as the standard deviation of QT-interval durations. QTV parameters that normalize for heart rate variability, like the QTV index [1], would require additional modeling of variations in RR-interval duration. The approach could also be applied to validate measurement algorithms of other types of variability, such as T-wave alternans, after appropriate modelling.

Our study has several limitations. First, our simulation of QTV by shifting the tail of individual T waves, preserving their shape, is straightforward but may not fully reflect reality. Unfortunately, little is known about the underlying mechanisms that affect QTV and the shape of the T wave. Once such knowledge becomes available, a more elaborate simulation is imaginable. Second, for practical reasons we only tested the effect of a limited set of artifacts, i.e., noise and residual baseline wander, but simulation of other types of artifacts can be envisaged. For example, simulated respiratory modulation of T-wave amplitudes has previously been shown to affect QTV estimates based on single-lead measurement [4]. Although we expect our algorithms to be less sensitive for respiratory movements because we combine information from all ECG leads, this may be investigated in future research.

In conclusion, artificially constructed ECGs with a variety of disturbances allow validation of QTV measurement procedures. The FSA algorithm provides accurate STV estimates under varying signal conditions, and performs significantly better than traditional beat-by-beat analysis. The fully automatic operation of the FSA algorithm enables STV measurement in large sets of ECGs.
